# Reward-Related Behavioral, Neurochemical and Electrophysiological Changes in a Rat Model of Autism Based on Prenatal Exposure to Valproic Acid

**DOI:** 10.3389/fncel.2019.00479

**Published:** 2019-10-25

**Authors:** Sara Schiavi, Daniela Iezzi, Antonia Manduca, Stefano Leone, Francesca Melancia, Carmen Carbone, Michele Petrella, Guido Mannaioni, Alessio Masi, Viviana Trezza

**Affiliations:** ^1^Department of Science, Section of Biomedical Sciences and Technologies, University “Roma Tre”, Rome, Italy; ^2^Department of Neuroscience, Psychology, Drug Research and Child Health -NEUROFARBA-, Section of Pharmacology and Toxicology, School of Psychology, University of Florence, Florence, Italy; ^3^School of Pharmacy, University of Camerino, Camerino, Italy

**Keywords:** autism, valproate, social play behavior, dopamine, electrophysiology

## Abstract

Prenatal exposure to the antiepileptic drug valproic acid (VPA) induces autism spectrum disorder (ASD) in humans and autistic-like behaviors in rodents, which makes it a good model to study the neural underpinnings of ASD. Rats prenatally exposed to VPA show profound deficits in the social domain. The altered social behavior displayed by VPA-exposed rats may be due to either a deficit in social reward processing or to a more general inability to properly understand and respond to social signals. To address this issue, we performed behavioral, electrophysiological and neurochemical experiments and tested the involvement of the brain reward system in the social dysfunctions displayed by rats prenatally exposed to VPA (500 mg/kg). We found that, compared to control animals, VPA-exposed rats showed reduced play responsiveness together with impaired sociability in the three-chamber test and altered social discrimination abilities. In addition, VPA-exposed rats showed altered expression of dopamine receptors together with inherent hyperexcitability of medium spiny neurons (MSNs) in the nucleus accumbens (NAc). However, when tested for socially-induced conditioned place preference, locomotor response to amphetamine and sucrose preference, control and VPA-exposed rats performed similarly, indicating normal responses to social, drug and food rewards. On the basis of the results obtained, we hypothesize that social dysfunctions displayed by VPA-exposed rats are more likely caused by alterations in cognitive aspects of the social interaction, such as the interpretation and reciprocation of social stimuli and/or the ability to adjust the social behavior of the individual to the changing circumstances in the social and physical environment, rather than to inability to enjoy the pleasurable aspects of the social interaction. The observed neurochemical and electrophysiological alterations in the NAc may contribute to the inability of VPA-exposed rats to process and respond to social cues, or, alternatively, represent a compensatory mechanism towards VPA-induced neurodevelopmental insults.

## Introduction

Although the precise causes of autism spectrum disorder (ASD) are still the subject of significant debate, a number of factors (rare gene mutations, gene variations and adverse environmental events) have been identified that, interacting in complex ways, affect early brain development contributing to the risk of ASD. Among the environmental factors involved in the pathogenesis of ASD, it has been well documented that prenatal exposure to the antiepileptic drug valproic acid (VPA) is associated with increased risk of neurodevelopmental delay and autistic symptoms in the offspring. Indeed, when given during gestation, VPA not only increases the risk for various congenital malformations (Kozma, 2001; Kini et al., 2006), but also induces core autistic symptoms in the offspring, i.e., impaired communication, reduced sociability and stereotyped behaviors (Williams and Hersh, 1997; Williams et al., 2001). Based on this clinical evidence, prenatal exposure to VPA in rodents has been validated as a drug-induced preclinical model of ASD (Roullet et al., 2013; Nicolini and Fahnestock, 2018; Tartaglione et al., 2019). In agreement with the clinical data, rodents exposed to VPA during pregnancy show marked behavioral impairments resembling the core and secondary signs of ASD (Rodier et al., 1996; Narita et al., 2002; Miyazaki et al., 2005; Schneider and Przewłocki, 2005; Servadio et al., 2016; Melancia et al., 2018). Since impaired social interaction is a key feature of ASD, a valid animal model is expected to exhibit deficits in this behavioral domain. Rodents are highly social species that engage in complex patterns of social behavior such as parental care and social play behavior (Panksepp et al., 1984; Ricceri et al., 2007). Rats prenatally exposed to VPA show a wide range of deficits in the social domain. At infancy, they show deficits in social communication and social discrimination, i.e., they are unable to properly communicate with their mother and siblings when removed from the nest and cannot discriminate between a neutral scent and their own nest odor (Schneider and Przewłocki, 2005; Dufour-Rainfray et al., 2010; Favre et al., 2013; Servadio et al., 2016; Bronzuoli et al., 2018; Cartocci et al., 2018; Melancia et al., 2018). At adolescence, VPA-exposed rats display atypical patterns of social play behavior, that is the most characteristic and rewarding social activity displayed by young mammals: indeed, compared to control animals, rats prenatally exposed to VPA respond to play solicitation mainly by partial rotation and evasion, rather than reciprocating the playful interaction (Servadio et al., 2016; Melancia et al., 2018). The social deficits displayed by VPA-exposed rats are long lasting, since they also persist at adulthood (Schneider and Przewłocki, 2005; Schneider et al., 2006, 2008; Markram et al., 2008; Kim et al., 2011, 2014; Servadio et al., 2016, 2018; Hirsch et al., 2018; Melancia et al., 2018; Fontes-Dutra et al., 2019) and are evocative of the social disturbances displayed by autistic patients over the course of development.

The pervasive social deficits found in autistic patients have been initially explained in terms of cognitive impairments and inability to infer others’ mental states. More recently, they have been related to blunted social reward processing, i.e., inability to enjoy and prolong reciprocal social interactions, which has been hypothesized to be the consequence of abnormal activity of the brain reward circuit in social contexts (Chevallier et al., 2012; Pellissier et al., 2018). Along this line, the social dysfunctions displayed by VPA-exposed rats may be caused by either their inability to properly understand and respond to social signals by the social partner or by a failure of their reward system to assign a positive value to the social experience. The aim of the present study was to address this issue by performing behavioral, neurochemical and electrophysiological experiments in rats prenatally exposed to VPA. In particular, we determined whether the social deficits displayed by VPA-exposed rats are associated with changes in more specific reward-related behaviors, including social, drug and food rewards. Furthermore, given the important role of corticolimbic dopamine in (social) reward processes (Gunaydin et al., 2014; Vanderschuren et al., 2016), we measured the expression of D1 and D2 dopamine receptors in the prefrontal cortex (PFC), dorsal striatum (DS), nucleus accumbens (NAc) and hippocampus (HIPP) of VPA-exposed rats, since these brain areas play an important role in the modulation of social behavior. Last, since activation of dopaminergic terminals in the NAc of rats during bouts of interaction with novel conspecifics has been reported (Robinson et al., 2002; Gunaydin et al., 2014) and given the important role of NAc dopamine in rewarding forms of social interaction such as social play (Manduca et al., 2016), we addressed the role of the NAc in the social impairment displayed by VPA-exposed rats by performing electrophysiological experiments in this brain area.

## Materials and Methods

### Animals

Female Wistar rats (Charles River, Italy), weighing 250 ± 15 g, were mated overnight. The morning when spermatozoa were found was designated as gestational day 1. Pregnant rats were singly housed in Macrolon cages [40 (length) × 26 (width) × 20 (height) cm], under controlled conditions (temperature 20–21°C, 55%–65% relative humidity and 12/12 h light cycle with lights on at 07:00 h). Food and water were available *ad libitum*. On gestational day 12.5, females received a single intraperitoneal injection of either VPA or saline (SAL). Newborn litters found up to 17:00 h were considered to be born on that day [postnatal day (PND) 0]. On PND 1, the litters were culled to eight animals (six males and two females), to reduce the litter size-induced variability in the growth and development of pups during the postnatal period. On PND 21, the pups were weaned and housed in groups of three. The experiments were carried out on the male offspring during adolescence (PNDs 35–40) and adulthood (PNDs 90–95). One male pup per litter from different litters per treatment group was used in each experiment. For the flow cytometric experiments, we used brain samples from the VPA- and SAL-exposed rats tested in the social play behavior, the three-chamber and the social discrimination tests. Other cohorts of VPA- and SAL-exposed rats were used for the electrophysiology experiments and to investigate amphetamine-induced hyperlocomotion, sucrose preference and socially-induced Conditioned Place Preference (sCPP). The exact sample size (n) for each experimental group/condition is indicated in the figure legends. The sample size was based on our previous experiments and power analysis performed with the software G power.

The experiments were approved by the Italian Ministry of Health (Rome, Italy) and performed in agreement with the Animals in Research: Reporting *in vivo* Experiments (ARRIVE) guidelines (Kilkenny et al., 2010), with the guidelines released by the Italian Ministry of Health (D.L. 26/14) and the European Community Directive 2010/63/EU. In particular, the experimental protocol was approved by the Animal Care Committees of both Roma Tre and Florence Universities and by the Italian Ministry of Health (authorization numbers: 31-2019-PR and 955/2015-PR).

### Drugs

VPA (Cayman Chemical, Ann Arbor, MI, USA) was dissolved in saline at a concentration of 250 mg/ml and administered at a dose (500 mg/kg) and time (gestational day 12.5) that have been shown to induce autistic-like behavioral changes in the offspring (Servadio et al., 2016; Melancia et al., 2018). Amphetamine (AMPH, Research Biochemicals International) was dissolved in saline and administrated at the dose of 0.5 mg/kg 30 min before the open field test to both VPA- and SAL-exposed offspring. We used a dose of AMPH that is known to affect locomotor activity without inducing stereotyped behaviors (Fowler et al., 2003; Manduca et al., 2014).

### Behavioral Tests

#### Social Play Test

The test was performed in a sound-attenuated chamber under dim light conditions, as previously described (Trezza and Vanderschuren, 2008, 2009). At PNDs 35–40, rats were individually habituated to the test cage for 10 min on the 2 days before testing. On the test day, the animals were isolated for 3 h before testing. The test consisted of placing each experimental rat together with an untreated animal for 15 min in the testing chamber. In rats, a bout of social play behavior starts with one rat soliciting (“pouncing”) another animal, by attempting to nose or rub the nape of its neck. The animal that is pounced upon can respond in different ways: if the animal fully rotates to its dorsal surface, “pinning” is the result (one animal lying with its dorsal surface on the floor with the other animal standing over it), which is considered the most characteristic posture of social play behavior in rats. The following parameters were scored for each animal of a pair using the Observer 3.0 software (Noldus, The Netherlands): (1) number of pinning events; (2) number of pouncing events; (3) evasion (the animal that is pounced upon does not prolong the playful interaction but rather runs away); and (4) play responsiveness [the percentage of response to play solicitation, as the probability of an animal of being pinned in response to pouncing by the stimulus partner (Servadio et al., 2016)]. Time spent in social exploration (the total amount of time spent in non-playful forms of social interaction, like sniffing any part of the body of the test partner, including the anogenital area, or grooming any part of the partner body).

#### Three-Chambers Test

The test was performed as previously described (Servadio et al., 2016). The apparatus was a rectangular three-chamber box, with two lateral chambers (30 × 35 × 35 cm; l × w × h) connected to a central chamber (15 × 35 × 35 cm; l × w × h). Each lateral chamber contained a small Plexiglas cylindrical cage. At PND 90, each experimental rat was individually allowed to explore the three-chamber apparatus for 10 min and then confined in the central compartment. An unfamiliar stimulus animal was confined in a cage located in one chamber of the apparatus, while the cage in the other chamber was left empty. Both doors to the side chambers were then opened, allowing the experimental animal to explore the apparatus for 10 min. The discrimination index was scored using the Observer 3.0 software (Noldus, The Netherlands) and was calculated as the difference in time spent sniffing the stimulus animal and the time spent exploring the empty chamber, expressed as the percentage ratio of the total time spent exploring both the stimulus animal and the empty chamber.

#### Social Discrimination Test

The test was performed as previously described (Melancia et al., 2018). Briefly, animals were isolated for 7 days before testing. The test consisted of a learning trial and a retrieval trial, which were separated by a 30 min intertrial interval. During the learning trial, a juvenile (30 days old) unfamiliar rat was introduced into the home cage of the experimental rat for 5 min. The time spent by the experimental rat investigating (sniffing, allogrooming and following) the juvenile was measured. Thirty-minutes after, the juvenile used in the learning trial was returned to the same adult’s cage together with a novel juvenile. The time spent by the adult exploring the novel and the familiar juveniles was monitored for 5 min. The discrimination index was calculated as the difference in time exploring the novel and the familiar animal, expressed as the percentage ratio of the total time spent exploring both animals (Campolongo et al., 2007).

#### Open Field Test

To assess whether adolescent and adult VPA- and SAL-exposed rats similarly responded to AMPH-induced hyperlocomotion, animals from both experimental groups were tested for horizontal locomotor activity in a squared box [40 (length) × 40 (width) × 60 (height) cm]. Each animal was placed in the central zone of the apparatus and allowed to explore for 30 min. Total locomotor activity (expressed as the frequency of crossings in the arena) was analyzed during the 30-min test session. After each session, the apparatus was cleaned with ethanol 70%.

#### Sucrose Preference Test

At both adolescence and adulthood, rats were tested for preference of a 2% sucrose solution, using a two-bottle choice procedure (Monteggia et al., 2007) with slight modifications. Subjects were housed singly for the 3 days of test. Rats were given two bottles, one of sucrose (2%) and one of tap water. Every 24 h the amount of sucrose and water consumed was evaluated. To prevent potential location preference of drinking, the position of the bottles was changed every 24 h. The preference for the sucrose solution was calculated as the percentage of sucrose solution ingested relative to the total amount of liquid consumed.

#### Socially-Induced Conditioned Place Preference

The sCPP test was performed at adolescence and adulthood as previously described (Wei et al., 2015). Briefly, rats were placed in an acrylic box [75 (length) × 35 (width) × 35 (height) cm], divided into two chambers by a clear acrylic wall with a small opening. Each chamber contained different types of autoclaved, novel bedding (Sanyx Bio Ultra litter and Padovan Sandy Litter), which differed in texture and shade (white vs. dark-brown). A 30-min preconditioning test was used to establish any baseline preference for either of the two types of novel bedding. Individual rats with strong preference for either type of bedding were excluded (typically, those that spent more than 1.5× time on one bedding over the other). The next day, each experimental animal was assigned to a social cage with cage-mates to be conditioned to one type of novel bedding for 24 h (CS+). Then, the experimental rat was moved to an isolated cage with the other type of bedding for 24 h (CS−). Bedding assignments were counterbalanced for an unbiased design. Animals were then tested alone for 30 min in the two-chambered box and the time spent in each chamber was calculated to determine post-conditioning preference for either type of bedding. Fresh bedding was used at each step and chambers were thoroughly cleaned between sessions with ethanol 70% to avoid olfactory confounders.

### Brain Samples Collection

Rats were rapidly decapitated, and their brains were removed and cut into coronal slices on a cold plate. The PFC, DS, NAc and hippocampus (HIPP) were dissected by hand under microscopic control within 2 min as previously described (Hill et al., 2010; Trezza et al., 2012; Gray et al., 2015).

### Flow Cytometric Experiments

Brain samples for each brain area were transferred with PBS into 1.5 ml tube. After centrifugation, the pellet was digested with Trypsin (0.1%) for 30 min at 37°C on slight agitation. Cell suspensions were filtered with CellTrics (100 μm) and washed with 5 ml of PBS. A little sample of suspension was analyzed for forward and side scatter parameter for neuron population quality control (Cruz et al., 2013). Each sample was fixed with PFA (1%), permeabilized with ice-cold ethanol (70%) and incubated for at least 2 h at −20°C. Next, cells were centrifuged, rehydrated with 1 ml PBS/BSA (1%)/Triton (0.1%), aliquoted in 96 well tissue culture plates with conical bottom. Samples were incubated for 1 h RT with primary antibody anti-D1 (1:100 diluted, Novusbio AB81296) and D2 dopamine receptors (1:100 diluted, Santa Cruz SC-7523). The cells were washed and incubated with secondary antibody anti-rabbit and anti-goat Alexa 488 conjugated. All samples were counterstained with propidium iodide/RNase buffer for nuclei identification (with G0 cell cycle phase DNA content) and for singlet/doublets discrimination. Mean fluorescence intensity of 20,000 useful cellular events for each sample, was calculated.

### Whole-Cell Patch Clamp Recordings in Acute Brain Slices

Preparation of acute brain slices was performed with established procedures (Carbone et al., 2017). In brief, 1-month old male Wistar rats were anesthetized with isoflurane and decapitated. The brain was quickly removed and glued to the bottom of a vibroslicer slicing chamber (Leica VT 1000S, Leica Microsystem, Wetzlar, Germany). Coronal brain slices (250 μm) containing the NAc were cut in a slicing solution composed of (in mM): N-methyl-D-glucamine (92), 4-(2-hydroxyethyl)-1-piperazine-1-ethanesulfonic acid (20), glucose (25), NaHCO_3_ (30), NaH_2_PO_4_ (1.25), KCl (2.5), MgSO_4_ (10), CaCl_2_ (0.5). During slicing, the solution was kept cold and infused with a 95% O_2_ + 5% CO_2_ gas mixture. Slices were transferred to a warm (34–35°C), carbo-oxygenated recovery bath containing artificial Cerebral Spinal Fluid (aCSF) of the following composition (in mM): NaCl (130), KCl (3.5), NaH_2_PO_4_ (1.25), NaHCO_3_ (25), glucose (10), CaCl_2_ (2) and MgSO_4_ (1). Slices were allowed to recover for at least 30 min prior to experiments. During recordings, a single slice was kept in a flow chamber positioned under the microscope objective and continuously perfused with warm (34–35°C), carbo-oxygenated aCSF. Whole-cell pipettes were pulled from thin-walled borosilicate capillaries (Harvard Apparatus, London, UK) with a vertical puller (Narishige PP830, Narishige International Limited, London, UK) and back-filled with an intracellular solution containing (in mM): K+ methanesulfonate (120), KCl (15), HEPES (10), EGTA (0.1), MgCl_2_ (2), Na_2_Phosphocreatine (5), Na_2_GTP (0.3) and MgATP (2), resulting in a bath resistance of 3–5 MΩ. Unless otherwise specified, all drugs were purchased from Sigma-Aldrich (St. Louis, MO, USA) and bath-applied. Access resistance was monitored during voltage clamp recordings with 100-ms, −10-mV steps, throughout the experiment. Recordings undergoing a drift in access resistance ≥ 10% were discarded. No whole-cell compensations were used. Signals were sampled at 10 kHz and low-pass filtered at 3 kHz with an Axon Multiclamp 700B (Molecular Devices, Sunnyvale, CA, USA). NAc-MSNs were identified by their morphological and electrophysiological properties (Cepeda et al., 2008). To examine the effects of VPA prenatal exposure on medium spiny neuron (MSN) excitability, current clamp input-output curves were obtained by injecting 800 ms-current steps with amplitude ranging from −50 to +550 pA with 50 pA increments. Inwardly rectifying potassium currents (IKir) were obtained by imposing 500 ms-voltage steps with amplitude ranging from −150 to −60 mV with 10 mV increments. Potassium currents were recorded in the presence of tetrodotoxin (TTX, 1 μM, Tocris, Biosciences, Bristol, UK), a blocker of voltage-dependent sodium channels. IKir was isolated as the 1 mM CsCl-sensitive component. IKir current density was obtained by normalizing current amplitudes for membrane capacitance (derived from the area underlying current peaks elicited by −10 mV steps) and expressed as pA/pF.

### Statistical Analysis

Behavioral and neurochemical data are expressed as mean ± SEM. To assess the effects of the prenatal treatment (VPA or SAL) on the parameters measured, data were analyzed with Student’s *t*-tests. Two-way analysis of variance (ANOVA) was used to assess the effects of prenatal and postnatal treatments in the open field test, using prenatal (VPA or SAL) and postnatal (AMPH or vehicle) treatments as between-subjects factor. Two-way ANOVA was followed by Student’s–Newman–Keuls *post hoc* test where appropriate. All behavioral tests were scored by a trained observer who was unaware of treatment condition to reduce performance bias. Random allocation of animals to treatment groups and to behavioral tasks and blinding of investigators assessing outcomes were adopted to reduce selection and detection bias. All behavioral data were tested for normality.

For electrophysiological experiments, data are presented as mean ± SEM of *n* cells obtained from N animals. Statistical significance was assessed with student’s *t*-test for unpaired samples (Microcal Origin 9.1; Northampton, MA, USA). Significance at the *P* < 0.05, 0.01 and 0.001 level is indicated with *, ** and ***, respectively, in figures. Graphs and representative traces were generated with Microcal Origin 9.1. Example traces represent typical observations.

## Results

### Social Play Test

**Figure 1:**
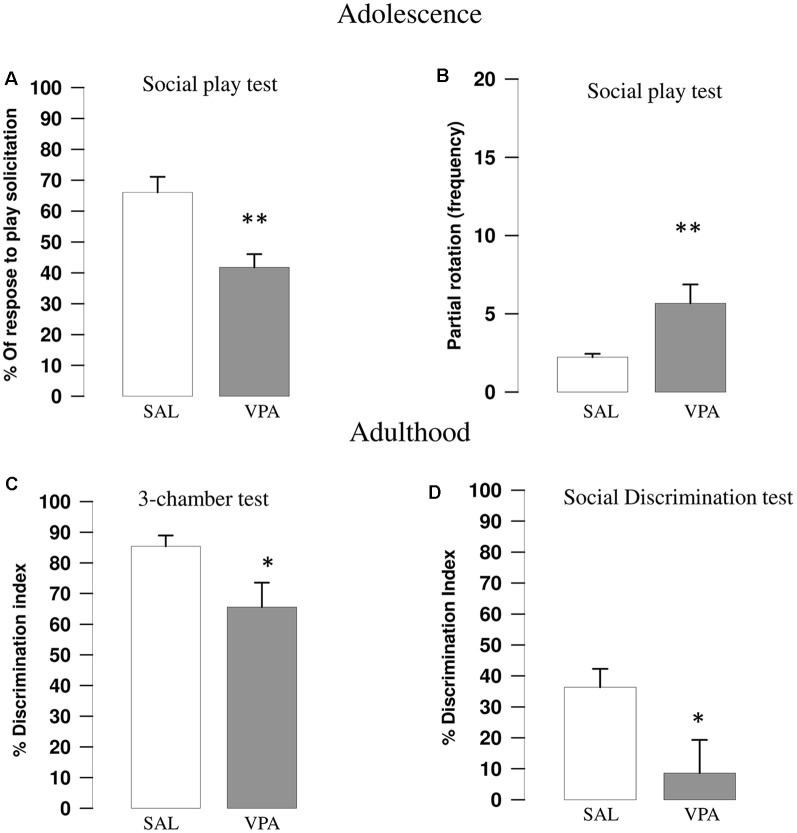
Valproic acid (VPA)-exposed rats showed altered social play behavior, impaired sociability in the three-chamber test and reduced social discrimination abilities. VPA-exposed rats showed reduced response to play solicitation **(A)** and increased frequency of partial rotation (**B**; *n* = SAL 9, *n* = VPA 9). VPA-exposed rats also showed reduced sociability in the three-chamber test **(C)**, since they showed a lower discrimination index indicating (i.e., they spent less time sniffing the stimulus animal compared to SAL-exposed rats; *n* = SAL 8, *n* = VPA 8). VPA-exposed rats also showed impaired social discrimination as they displayed a lower discrimination index compared to SAL-exposed animals in the social discrimination task (**D**; *n* = SAL 8, *n* = VPA 8). Data represent mean values ± SEM; *p < 0.05, **p < 0.01, vs. SAL group (Student’s *t*-test).

VPA-exposed rats showed reduced play responsiveness compared with SAL-exposed animals (*t* = 3.67, *p* = 0.002, *df* = 16; Figure 1A). Indeed, while no differences were found between SAL- and VPA-exposed animals in the number of pinning (*t* = 0.81, *p* = n.s., *df* = 16; data not shown) and pouncing (*t* = −0.86, *p* = n.s., *df* = 16; data not shown), VPA-exposed rats displayed a higher frequency of partial rotation (*t* = −2.81, *p* = 0.013, *df* = 16; Figure 1B) compared to SAL-exposed animals. No differences in the total time spent in general social exploration were found between SAL- and VPA-exposed animals (*t* = 1.43, *p* = n.s., *df* = 16, data not shown).

### Three-Chambers Test

VPA-exposed rats showed decreased sociability in the three-chamber test, as they spent less time sniffing the stimulus animal compared to SAL-exposed rats, showing a lower discrimination index (*t* = 2.27, *p* = 0.039, *df* = 14; Figure 1C).

### Social Discrimination Test

VPA-exposed animals showed impaired social discriminative abilities as they showed a lower discrimination index in the social discrimination test compared with SAL-exposed animals (*t* = 2.27, *p* = 0.044, *df* = 14; Figure 1D).

### Amphetamine-Induced Hyperlocomotion

**Figure 2:**
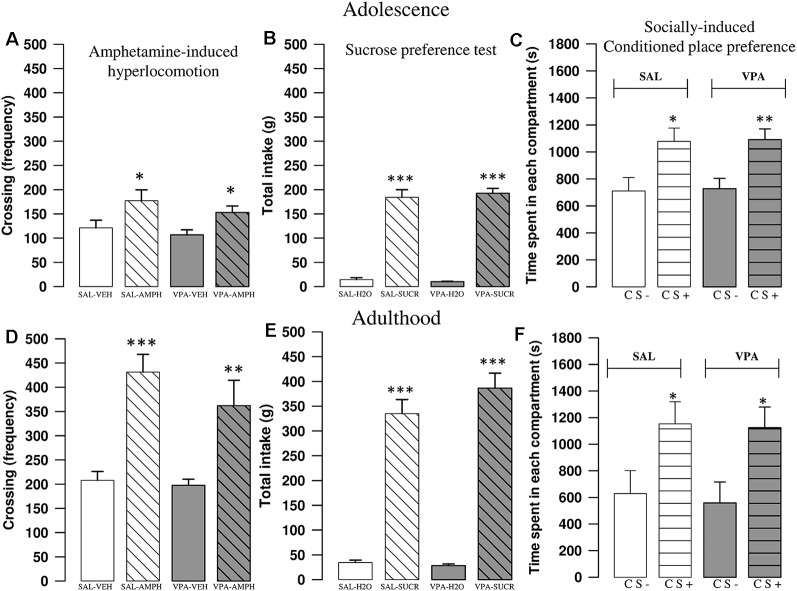
VPA-exposed rats did not differ from control rats in AMPH-induced hyperlocomotion, sucrose preference and socially-induced Conditioned Place Preference (sCPP). In the open field test, AMPH increased the frequency of crossing both in SAL- and VPA-exposed rats at postnatal days (PNDs) 30–35 (**A**; *n* = SAL-VEH 7, *n* = SAL-AMPH 7, *n* = VPA-VEH 9, *n* = VPA-AMPH 9) and PNDs 90–95 (**D**; *n* = SAL-VEH 8, *n* = SAL-AMPH 7, *n* = VPA-VEH 8, *n* = VPA-AMPH 8). Data represent mean values ± SEM; *p < 0.05, **p < 0.01, ***p < 0.001 vs. SAL-VEH group (Student-Newman-Keuls *post hoc* test). No differences were found between SAL- and VPA-exposed rats in the sucrose preference test, both at PNDs 30–35 (**B**; *n* = SAL 10, *n* = VPA 10) and PNDs 90–95 (**E**; *n* = SAL 10, *n* = VPA 10). Data represent mean values ± SEM; *** *p* < 0.001 vs. SAL-H_2_O and vs. VPA-H_2_O groups (student’s *t*-test). In the sCPP test, both SAL- and VPA-exposed rats spent more time in the chamber containing the bedding where social interaction previously occurred (CS+), both at PNDs 30–35 (**C**; *n* = SAL 12, *n* = VPA 12) and PNDs 90–95 (**F**; *n* = SAL 8, *n* = VPA 11). Data represent mean values ± SEM; *p < 0.05, **p < 0.01, vs. SAL-CS- and VPA-CS- groups (Student’s *t*-test).

AMPH increased locomotor activity of SAL- and VPA-exposed rats both at adolescence and adulthood. A two-way ANOVA analysis performed on the frequency of crossing after treatment with AMPH or its vehicle gave the following results: PNDs 35–40 (*F*_(prenatal treat.)1, 28_ = 1.56, *p* = n.s.; *F*_(treat.)1, 28_ = 10.97, *p* < 0.01; *F*_(prenatal treat. × treat.)1, 28_ = 0.1; *p* = n.s.); PNDs 90–95 (*F*_(prenatal treat.)1, 27_ = 1.40, *p* = n.s.; *F*_(treat.)1, 27_ = 33.05, *p* < 0.001; *F*_(prenatal treat. × treat.)1, 27_ = 0.76; *p* = n.s.). *Post hoc* analysis revealed that AMPH increased the frequency of crossing both in SAL- (PNDs 35–40: *p* < 0.05; PNDs 90–95: *p* < 0.001) and VPA-exposed rats (PNDs 35–40: *p* < 0.05; PNDs 90–95: *p* = 0.002; Figures 2A–D).

### Sucrose Preference Test

Both SAL- and VPA-exposed rats preferred the sucrose over the water solution the in the sucrose preference test, at both PNDs 35–40 and 90–95 (PNDs 35–40: SAL: *t* = −10.53, *p* < 0.001, df = 16; VPA: *t* = −17.65, *p* < 0.001, *df* = 12, Figure 2B; PNDs 90–95: SAL: *t* = −10.43, *p* < 0.001, *df* = 18; VPA: *t* = −11.76, *p* < 0.001, *df* = 18; Figure 2E).

### Socially-Induced Conditioned Place Preference

Both at adolescence and adulthood, VPA-exposed rats did not show deficits in the sCPP test. Indeed, during the test session animals of both experimental groups spent more time in the chamber containing the bedding used for the social conditioning (CS+; PNDs 35–40: Saline group: *t* = −2.63, *p* < 0.05, *df* = 22; VPA group: *t* = −3.31, *p* < 0.01, *df* = 22; Figure 2C; PNDs 90–95: Saline group: *t* = −2.19, *p* < 0.05, *df* = 14; VPA group: *t* = −2.55, *p* < 0.05, *df* = 20; Figure 2F).

### Flow Cytometric Analysis of Dopamine Receptors

**Figure 3:**
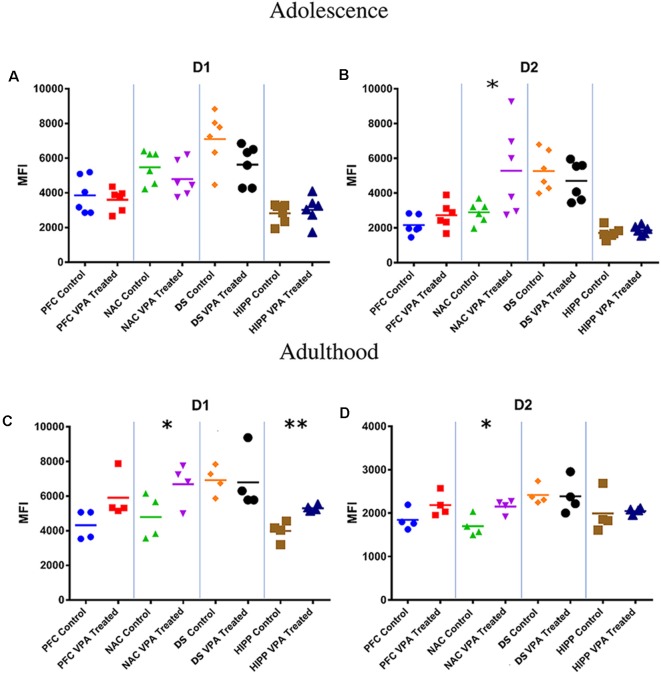
Flow cytometric analysis of dopamine receptors. No differences between VPA-exposed and SAL-exposed rats were found in D1 dopamine receptor expression in prefrontal cortex, nucleus accumbens, dorsal striatum and hippocampus **(A)**. At PNDs 30–35, VPA-exposed rats showed a significant increase in D2 dopamine receptor expression in the nucleus accumbens (NAc) **(B)** compared to SAL-exposed rats (*n* = SAL 6, *n* = VPA 6). At PNDs 90–95, VPA-exposed rats showed increased expression of D1 dopamine receptor expression in NAc and hippocampus **(C)**, while D2 receptor expression was increased only in the NAc (**D**; *n* = SAL 5, *n* = VPA 5). Data represent mean values ± SEM; *p < 0.05, **p < 0.01 vs. SAL group (Student’s *t*-test).

At adolescence, VPA-exposed rats shows a significative increase in D2 dopamine receptor expression in the NAc (SAL-exposed animals: MFI = 2,891; VPA-exposed animals: MFI = 5,272, *p* < 0.05; Figures 3A,B).

At adulthood, VPA-exposed rats showed a significative increase in D1 dopamine receptor expression in the NAc (SAL-exposed animals: MFI = 4,789; VPA-exposed animals: MFI = 6,690, *p* = 0.025; Figure 3C) and HIPP (SAL-exposed animals: MFI = 3,987; VPA group: MFI = 5,299, *p* = 0.008; Figure 3C), whereas D2 dopamine receptors were significative increased only in the NAc (SAL-exposed animals: MFI = 1,697; VPA-exposed animals: MFI = 2,152, *p* = 0.022; Figure 3D).

### Electrophysiological Recordings of NAc MSNs in Acute Brain slices

**Figure 4:**
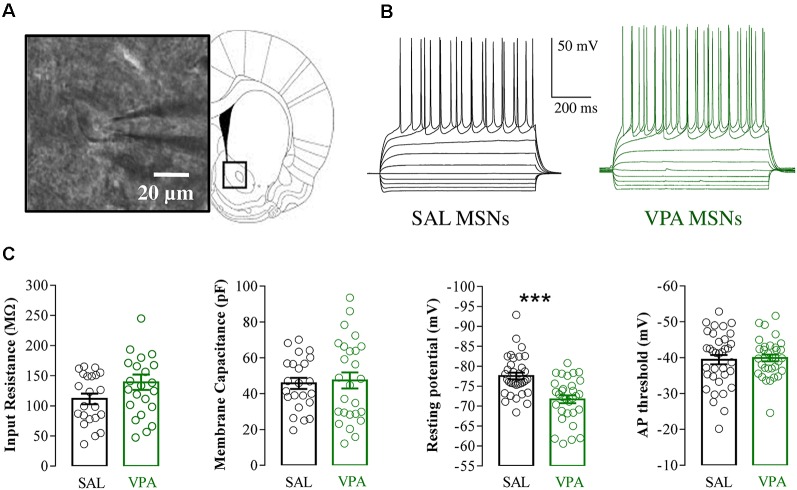
Electrophysiological properties of SAL medium spiny neurons (MSNs) and VPA MSNs.** (A)** NAc-MSN visualized with infrared video microscopy (*left*) and schematic representation of a coronal brain slice at the level of the NAc (*right*). **(B)** Representative voltage traces of SAL MSNs and VPA MSNs evoked by injection of hyperpolarizing and depolarizing current pulses (800 ms from −200 pA to 250 pA in 50 pA steps). **(C)** Passive membrane properties of SAL MSNs and VPA MSNs; input resistance (SAL MSNs 111.4 ± 8.81 MΩ, *n* = 22; VPA MSNs 139.2 ± 12.78 MΩ, *n* = 23), membrane capacitance (SAL MSNs 45.73 ± 3.07 pF *n* = 23; VPA MSNs 47.36 ± 4.49 pF, *n* = 26); resting membrane potential (SAL MSNs −77.59 ± 0.83 mV, *n* = 35; VPA MSNs −71.72 ± 0.98 mV, *n* = 37) and AP threshold (SAL MSNs −39.43 ± 1.3 mV, *n* = 35; VPA MSNs −39.96 ± 0.93 mV, *n* = 33). Data represent mean values ± SEM; ***p < 0.001 vs. SAL group (Student’s *t*-test).

**Figure 5:**
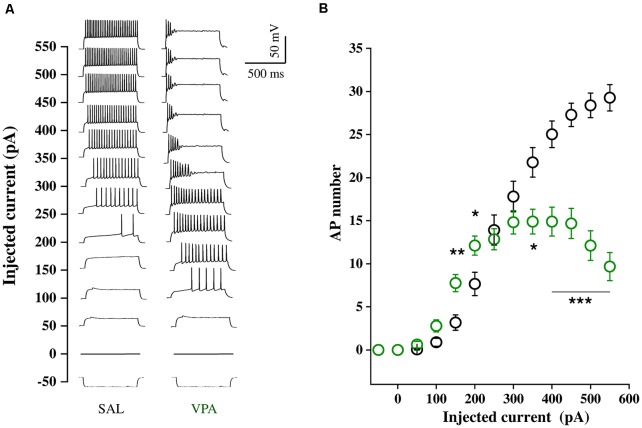
Intrinsic excitability of SAL MSNs and VPA MSNs.** (A)** Representative voltage traces obtained in response to–50 to 550 pA current steps (800 ms duration, 50 pA amplitude) from resting potential, recorded from SAL and VPA MSNs. **(B)** Number of APs fired plotted against current steps of increasing value (SAL MSNs, *n* = 35; VPA MSNs, *n* = 37). Data represent mean values ± SEM; *p < 0.05, **p < 0.01, ***p < 0.001 vs. SAL group (Student’s *t*-test).

**Figure 6:**
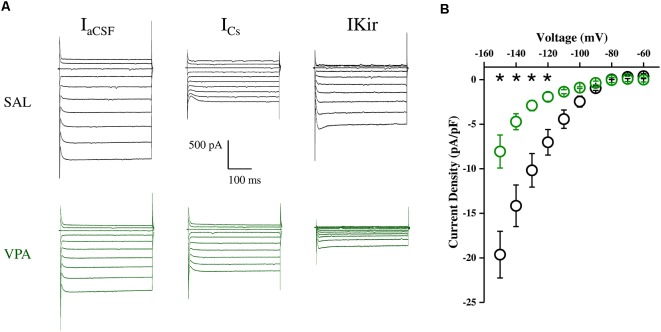
Inwardly rectifying potassium current (IKir) density in SAL MSNs and VPA MSNs.** (A)** Representative IKir traces obtained at hyperpolarized membrane potentials (−60 to −150 mV in 10 mV steps) obtained from SAL and VPA MSNs. IKir is obtained by subtracting I_Cs_ from I_aCSF_. Current-voltage relationship of IKir current density in SAL and VPA MSNs. **(B)** Current-voltage relationship of IKir current density in SAL and VPA MSNs (SAL MSNs, *n* = 7; VPA MSNs *n* = 5). Data represent mean values ± SEM; *p < 0.05 vs. SAL group (Student’s *t*-test).

Whole cell patch clamp recordings were obtained from NAc MSNs in acute coronal slices prepared from SAL- and VPA-exposed rats at PNDs 30–35 (*N* = 9 and 7, respectively, Figures 4A,B). While we found no differences in passive membrane properties between MSNs from SAL-exposed rats (SAL MSNs) and VPA-exposed rats (VPA MSNs; input resistance: SAL MSNs = 111.4 ± 8.81 MΩ, *n* = 22; VPA MSNs = 139.2 ± 12.78 MΩ, *n* = 23; membrane capacitance: SAL MSNs = 45.73 ± 3.07 pF, *n* = 23; VPA MSNs = 47.36 ± 4.49 pF *n* = 26; threshold: SAL MSNs = −39.43 ± 1.3 mV, *n* = 35; VPA MSNs = −39.96 ± 0.93 mV, *n* = 33; *p* = n.s.; all; Figure 4C), the latter group showed a significant depolarization of the resting membrane potential as compared to SAL MSNs (SAL MSNs = −77.59 ± 0.83 mV, *n* = 35; VPA MSNs = −71.72 ± 0.98 mV, *n* = 37; *p* = 0.0008; Figure 4C). We then determined the intrinsic excitability of MSNs in acute slices containing the NAc by measuring the number of action potentials elicited by depolarizing current steps of increasing amplitude. Figure 5A shows a direct comparison of representative voltage responses obtained at each value of imposed current from SAL and VPA MSNs. The minimal value of current amplitude required to elicit an action potential in VPA MSNs was significantly lower compared to SAL MSNs (100 pA vs. 200 pA). However, as the amplitude of the depolarizing current increased (≥250 pA), while the number of APs fired by SAL MSNs increased linearly, VPA MSNs progressively lost the ability to fire action potentials. The plot in Figure 5B reports the mean number of APs ± SEM fired by SAL (black) and VPA (green) MSNs vs. imposed current (SAL MSNs, *n* = 35, vs. VPA MSNs *n* = 37; 150 pA; *p* = 0.01; 200 pA; *p* = 0.03; 350 pA; *p* = 0.02; ≥ 400 pA; *p* < 0.0001). Depolarized resting potential and altered AP discharge pattern are consistent with a change in the density of whole-cell K^+^ currents. Based on the reported relevance of the inward-rectifying K^+^ current (IKir) in setting resting potential in MSNs (Kreitzer, 2009), we measured whole-cell currents elicited at hyperpolarizing potentials in control aCSF and in the presence of 1 mM Cs^+^, a IKir blocker (Cazorla et al., 2012). Figure 6A shows representative inward currents obtained in control solution (I_aCSF_) and 1 mM Cs^+^ (I_Cs_). IKir is obtained by subtracting I_Cs_ from I_aCSF_. Current-voltage relationship reveals a significant reduction of IKir current density in VPA MSNs (SAL MSNs, *n* = 7, VPA MSNs, *n* = 5; *p* < 0.05 where indicated; Figure 6B).

## Discussion

Clinical studies have repeatedly reported that maternal use of VPA during pregnancy can induce a wide range of abnormalities in the exposed children, ranging from structural malformations to more subtle autistic-like behaviors. For this reason, prenatal VPA exposure is nowadays considered an environmental risk factor involved in the pathogenesis of ASD (Christensen et al., 2013; Nicolini and Fahnestock, 2018). Based on the robust clinical evidence, prenatal exposure to VPA in rodents has been validated as a drug-induced preclinical model of ASD (Roullet et al., 2013; Ranger and Ellenbroek, 2016; Tartaglione et al., 2019).

In the present study, we show that prenatal exposure to VPA causes selective deficits in the social domain in the rat offspring at different developmental periods, together with changes in D1 and D2 dopamine receptor expression in the NAc and hyperexcitability in this same brain area, but without inducing changes in social and non-social reward-related behaviors.

According to the DSM-5 diagnostic criteria, persistent deficits in social communication and social interaction are key features of ASD. Social play behavior has a crucial role in the identification and diagnosis of ASD (Jordan, 2003; Jarrold et al., 2013) Social play is the first form of non-mother directed social behavior displayed by most mammals at young age (Panksepp et al., 1984; Vanderschuren et al., 1997, 2016). In ASD, play patterns are characterized not only by deficient cognitive complexity but also by a typical asocial dimension. Since the opportunity to engage in social play is crucial to acquire proper social and cognitive skills (Vanderschuren et al., 2016; Nijhof et al., 2018), the lack of social play in children with ASD has deleterious effects on their development, leading to long lasting deficits in self-awareness, social competence, problem solving and behavioral flexibility. In line with the social deficits reported in previous studies (Schneider and Przewłocki, 2005; Schneider et al., 2006; Felix-Ortiz and Febo, 2012; Servadio et al., 2016; Melancia et al., 2018), we confirm here that adolescent rats prenatally exposed to VPA show decreased responsiveness to play solicitation, since they respond to play solicitation mainly by partial rotation than reciprocating the playful interaction. Notably, these social deficits were long lasting. Indeed, VPA exposed rats showed social deficits also when tested at adulthood in the three-chamber and social discrimination tests. These data corroborate our previous findings showing that prenatal VPA exposure induces in the rat offspring a wide range of social impairments in the course of development, ranging from social play deficits to altered sociability and social discrimination (Servadio et al., 2016, 2018; Melancia et al., 2018) and are in line with clinical observation that children, adolescents and adults with ASD demonstrate marked socio-communicative deficits (Lai et al., 2011; Dworzynski et al., 2012; Head et al., 2014).

As it has been hypothesized for autistic patients (Chevallier et al., 2012), the wide range of social dysfunctions displayed by VPA-exposed rats may be due to either their inability to properly percept, understand and respond to socially relevant cues, or to a failure of their brain reward system to assign a positive value to the social experience (Pellissier et al., 2018). To address this issue, we performed neurochemical and electrophysiological experiments focusing on brain areas involved in reward processing, and performed additional behavioral experiments to investigate if VPA-exposed rats differ from control animals in responding to social and other (non-social) rewarding stimuli.

Brain regions involved in the control of social behavior include corticolimbic structures and their altered functionality may represent one neural substrate contributing to the social impairments characteristic of ASD (Scott-Van Zeeland et al., 2010; Chevallier et al., 2012; Ameis and Catani, 2015; Supekar et al., 2018). Notably, these regions are subjected to modulation by dopaminergic neurons and it has been suggested that a dysfunction of dopaminergic neurotransmission in the mesocorticolimbic circuit leads to the social deficits observed in ASD (Pavăl et al., 2017): thus, it has been demonstrated that striatal MSNs show enriched expression of genes associated with ASD (Chang et al., 2015) and that ASD-associated mutations affect specific MSN subtypes (Portmann et al., 2014; Rothwell et al., 2014; for a review, see Rothwell, 2016).

In autistic subjects, dopamine imbalances, manifested as either hyperactivity or hypoactivity of midbrain dopaminergic pathways, have been detected, highlighting the heterogeneity of the disease (Pavăl et al., 2017).

Dopamine activation of D1 and D2 receptors in corticolimbic regions is also important for the expression of social behavior in rodents (Robinson et al., 2002; Gunaydin et al., 2014; Manduca et al., 2016; Kopec et al., 2018). Therefore, although the social impairments observed in VPA-exposed animals likely cannot be ascribed to the alteration of a single neurotransmitter system, we focused the biochemical and electrophysiological analyses on dopaminergic neurotransmission in corticolimbic brain areas. We measured the expression of D1 and D2 dopamine receptors in the PFC, DS, NAc and HIPP since these brain areas play not only an important role in the modulation of the rewarding properties of social interactions but have also a key role in cognitive aspects of the social repertoire (e.g., social cognition). While the DS and NAc have a well-recognized role in the modulation of several aspects of reward-related behaviors (for a review, see Bhanji and Delgado, 2014), the PFC and HIPP have also been found to be deeply involved in cognitive aspects of social behavior (Vanderschuren et al., 2016; Montagrin et al., 2018).

We found that, compared to control animals, adolescent rats prenatally exposed to VPA showed increased expression of D2 dopamine receptors in the NAc. At adulthood, they showed increased expression of D1 dopamine receptors in the NAc and hippocampus and of D2 receptors in the NAc. These results suggest that the social deficits displayed by VPA-exposed rats in the course of development may arise from dopaminergic dysfunctions in both these brain regions.

In line with our results, changes in hippocampal dopamine and D1 receptor levels associated with social deficits have been demonstrated in a genetic mouse model exhibiting autism-relevant behavioral abnormalities (Liu et al., 2017). Furthermore, mice knockout for genes strongly associated with ASD, such as Cntnap4 (Karayannis et al., 2014) and neuroligin-3 (Rothwell et al., 2014) mutant mice, show changes in NAc dopaminergic neurotransmission together with deficits in the core ASD behavioral domains. Recently, it has also been demonstrated that altered VTA dopamine neuron function represents a key mechanism by which insufficiency of SHANK3, encoding the synapse scaffolding protein SHANK3, generates impaired social preference in mice (Bariselli et al., 2016).

Building on this evidence, we studied the basic electrophysiological properties of NAc MSNs in acute striatal brain slices of VPA- and SAL-exposed offspring. We found that NAc MSNs of VPA-exposed animals show a significant depolarization of the resting membrane potential and increased excitability in the lower part of the excitability curve, indicating higher firing probability in conditions of normal synaptic excitation. These changes are likely caused by altered Kir current density, known to determine the hyperpolarized value of membrane potential in normal MSNs (~−85 mV) and to affect their AP discharge pattern (Kreitzer, 2009; Cazorla et al., 2012). The NAc is a major node of the mesolimbic dopaminergic system and the overall impact of NAc output on behavior depends on the relative activity of D1- vs. D2-expressing MSNs. Increased excitability of MSNs of VPA-exposed animals, combined with increased expression of D2R-expressing neurons, is consistent with an imbalance in the direct and indirect pathway activity, in favor of the latter. Proper activation of these pathways underlies proper motor learning as well as the acquisition of reward-related behaviors (Yawata et al., 2012; Shin et al., 2018). Furthermore, extensive alterations in normal gene expression pattern have been found in multiple striatal neuronal populations in the VPA model of ASD (Lauber et al., 2016).

Based on the evidence of neurochemical and electrophysiological changes in the brain reward system of VPA-exposed rats, we tested their behavioral response to different (i.e., social and non-social) rewarding stimuli. First, we tested whether VPA- and SAL-exposed rats differently responded to a dose of amphetamine known to induce hyperlocomotion (Bolanos et al., 1998), a proxy for the ability of the dopaminergic system to respond to pharmacological activation. We found a robust amphetamine-induced increase in motor activity in the open field test in both adolescent and adult VPA- and SAL-exposed animals, showing a same susceptibility to amphetamine-induced hyperlocomotion in both experimental groups.

To examine whether the social impairment displayed by VPA-exposed rats was accompanied by a generalized anhedonic behavior, that is, a reduction in the interest for natural reward, we performed a sucrose preference test. In line with previous studies performed with other preclinical models of ASD (Jamain et al., 2008; Radyushkin et al., 2009) and with clinical studies reporting intact hedonic responses to sweet taste in autistic patients (Damiano et al., 2014), we found that both VPA-exposed and control animals showed preference for the sucrose over the water solution, either at adolescence or adulthood.

To assess whether the social deficits displayed by VPA-exposed animals may be due to aberrant social reward processing, we tested VPA- and SAL-exposed rats in a socially-induced place-conditioning task (sCPP), which involves the association between a social stimulus and a distinct set of contextual cues (Trezza and Vanderschuren, 2009; Dölen et al., 2013; Wei et al., 2015). Attenuated sCPP has been demonstrated in genetic models of ASD such as in fmr1 mutant mice, a model for Fragile X syndrome (Pacey et al., 2011), and BTBR T+tf/J (BTBR) mice (Pearson et al., 2012). However, we here failed to find any difference in sCPP: thus, both VPA- and SAL-exposed animals spent more time during testing in the chamber associated with a bedding where social interaction previously occurred. Thus, the altered pattern of social play behavior, the reduced sociability and the impaired social discrimination abilities displayed by VPA-exposed rats are not accompanied by altered social reward processing.

## Limitations

From the behavioral point of view, the present study has some methodological limitations. Indeed, while we here found that VPA- and SAL-exposed rats similarly respond to amphetamine-induced hyperlocomotion, it still needs to be determined whether VPA- and SAL-exposed animals differ in behavioral set-ups specifically designed to assess drug intake and drug addiction. Similarly, we cannot exclude that VPA-exposed animals would show altered sucrose preference if different concentrations of sucrose solution were used, as found in the SHANK3 mouse model of ASD, where Shank3 mice preferred a sucrose solution at high but not at low concentrations (Bariselli et al., 2016). Last, it is still possible that VPA-exposed rats would show altered social motivation when tested in socially-driven operant conditioning paradigms (Achterberg et al., 2016). Concerning the electrophysiological experiments, the potential limitations in the validity of the information obtained with brain slice recordings is related to the nature of the methodology, which allows for in-depth, reliable interrogation of cell-autonomous electrical properties, but inevitably involves profound alteration of network connectivity, even within the area under examination. Aware of the intrinsic advantages and limitations of this approach, here we focused on neuronal properties that are better preserved in the acute brain slice preparation, such as intrinsic neuronal excitability.

## Conclusion

Although more levels of analysis are needed to shed light on the mechanisms underlying the aberrant behavior found in VPA-exposed rats, on the basis of the results obtained here we suggest that the reduced play responsiveness, the impaired sociability in the three-chamber test and the reduced discrimination abilities in the social discrimination tasks displayed by VPA-exposed animals reported in the present and in previous studies (Kim et al., 2011, 2013, 2014; Servadio et al., 2016; Melancia et al., 2018) are more likely due to changes in the cognitive functions required for proper social interaction, such as to understand and predict the behaviors of other conspecifics or to adapt the social behaviors of the animal to the changing circumstances in its social and physical environment, or may be due to impairments in aspects of reward processing that could not be detected with the behavioral tasks used in the present work. In this picture, the alteration in the expression of striatal dopamine receptors and in the electrical properties of MSNs in the NAc may be interpreted as a homeostatic mechanism deployed by reward-related brain areas to compensate for VPA-induced neurodevelopmental perturbations. Further behavioral, neurochemical and electrophysiological investigations are required to support this interpretation.

## Data Availability Statement

The datasets used and/or analyzed during the current study are available from the corresponding author on reasonable request.

## Ethics Statement

The experiments were approved by the Italian Ministry of Health (Rome, Italy) and performed in agreement with the ARRIVE (Animals in Research: Reporting *in vivo* Experiments; Kilkenny et al., 2010) guidelines, with the guidelines released by the Italian Ministry of Health (D.L. 26/14) and the European Community Directive 2010/63/EU. In particular, the experimental protocol was approved by the Animal Care Committees of both Roma Tre and Florence Universities and by the Italian Ministry of Health (authorization numbers: 31-2019-PR and 955/2015-PR).

## Author Contributions

SS and FM performed, analyzed and contributed to the design of the behavioral experiments. SL performed, analyzed and designed the flow cytometric experiments. DI, CC and MP performed, analyzed and contributed to the design of the electrophysiology experiments. AMan and GM contributed to the design of the experiments and edited the manuscript. SS and DI wrote the manuscript. VT and AMas supervised the project, designed the experiments and wrote the manuscript.

## Conflict of Interest

The authors declare that the research was conducted in the absence of any commercial or financial relationships that could be construed as a potential conflict of interest.
